# Supplementary material for: Antiviral HIV-1 SERINC restriction factors disrupt virus membrane asymmetry
Source: Nat Commun. 2023 Jul 20;14:4368. doi: 10.1038/s41467-023-39262-2 (PMC10359404; doi:10.1038/s41467-023-39262-2)

Supplementary Information

Antiviral HIV-1 SERINC Restriction Factors Disrupt Virus Membrane Asymmetry

Susan A. Leonhardt, Michael D. Purdy, Jonathan R. Grover, Ziwei Yang, Sandra Poulos, William E. McIntire, Elizabeth A. Tatham, Satchal K. Erramilli, Kamil Nosol, Kin Kui Lai, Shilei Ding, Maolin Lu, Pradeep D. Uchil, Andrés Finzi, Alan Rein, Anthony A. Kossiakoff, Walther Mothes and Mark Yeager

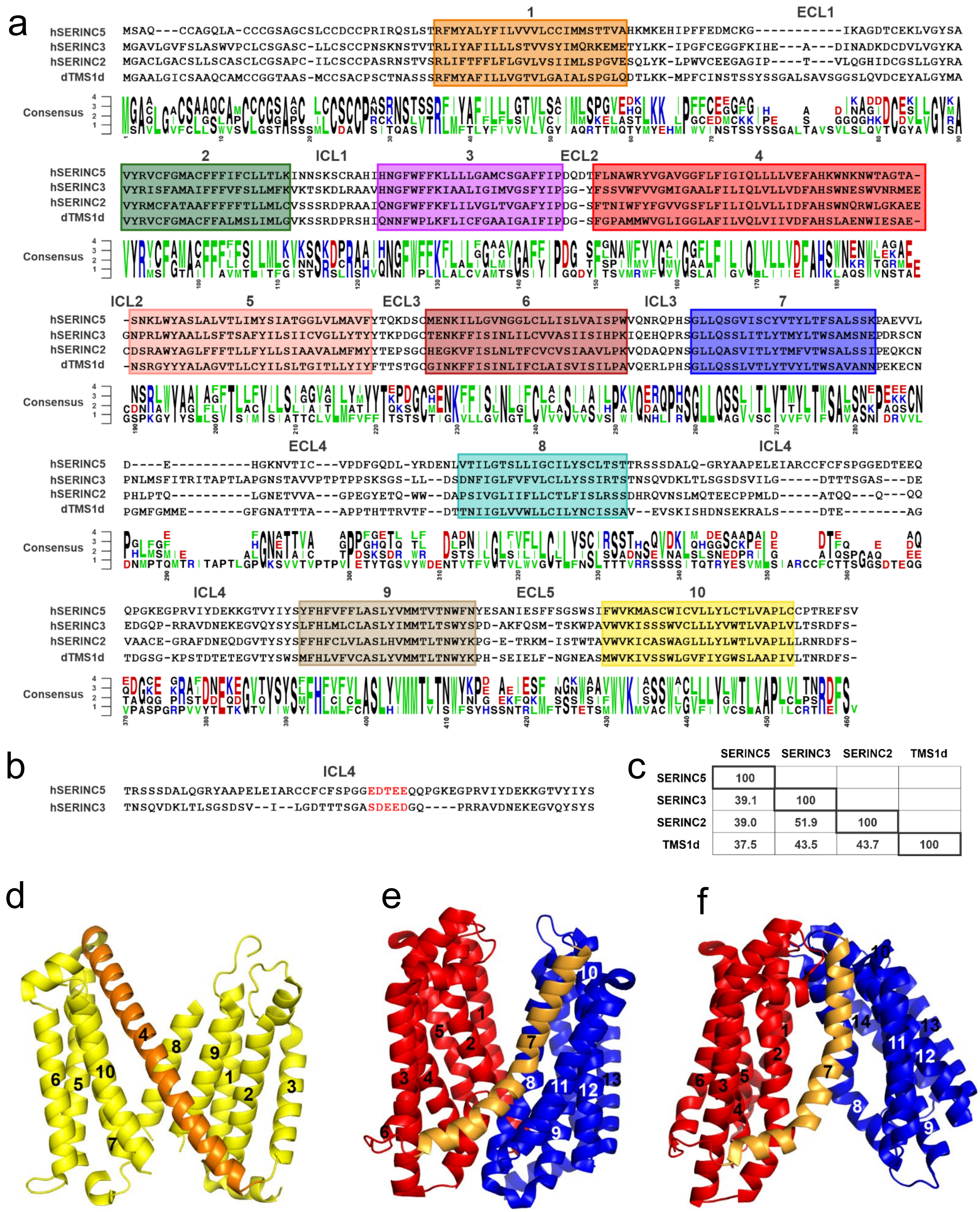

	SERINC5	SERINC3	SERINC2	TMS1d
SERINC5	100			
SERINC3	39.1	100		
SERINC2	39.0	51.9	100	
TMS1d	37.5	43.5	43.7	100

Supplementary Figure 1. | Sequence alignments and comparisons using Clustalω and architectural homology between hSERINC3 and the bacterial lipid II flippase MurJ. (a) The sequence alignments include hSERINCs 5 and 3 and a Drosophila ortholog (TMS1d), which display restriction activity, and hSERINC2, which does not display restriction activity. All four proteins display ten predicted transmembrane α-helices (H1-H10). The predicted intracellular (ICL) and extracellular (ECL) loops are indicated. **(b)** Alignment of the ICL4 regions from hSERINCs 5 and 3 from (a), highlighting the analogous acidic cluster motifs in red. **(c)** The Percent Identity matrix of hSERINCS5, 3 and 2 and TMS1d indicate 40-50% identity between the proteins. **(d)** Structure of SERINC3 (PDB: 7RU6 [http://10.2210/pdb7RU6/pdb]) colored yellow with crossmember (H4, orange) connecting the lobe comprised of helices 1, 2, 3, 8 and 9 with the lobe comprised of helices 5, 6, 7 and 10. Fragment of Fab bound is shown in cyan. **(e)** Outward open conformation of MurJ (PDB: 6NC9 [http://10.2210/pdb6NC9/pdb]) with analogous crossmember (helix 7, gold) connecting lobe comprised of helices 1-6 (red) with the lobe comprised of helices 8-14 (blue). Note that notwithstanding the homologous lobe architecture between Serinc3 and MurJ, the helical composition of the lobes is not conserved. **(f)** Inward open conformation of MurJ (PDB: 6NC7[http://10.2210/pdb6NC7/pdb]), colored as in **(b)**.

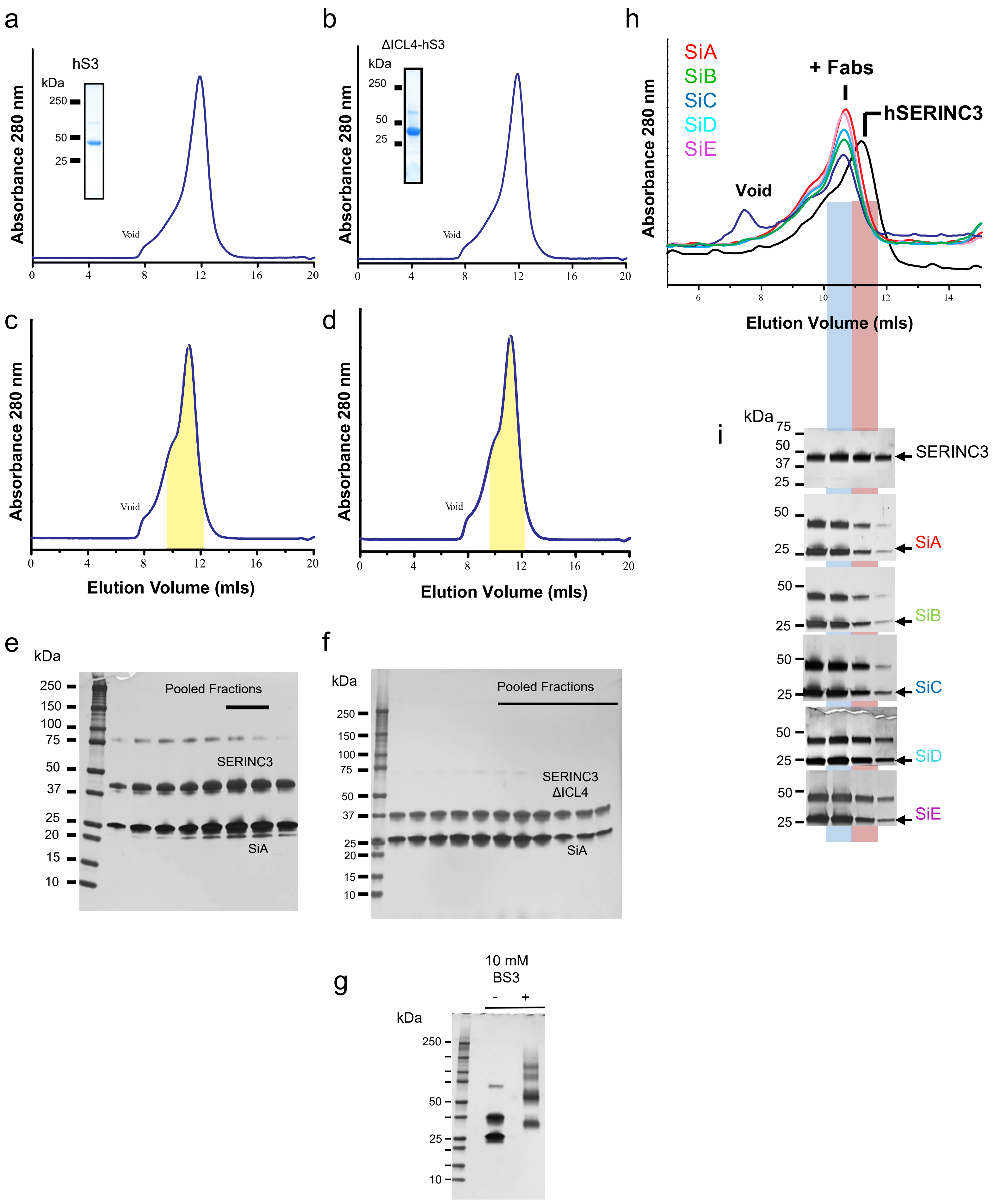

a
hS3
kDa
250
50
25
Void
Absorbance 280 nm
b
ΔICL4-hS3
kDa
250
50
25
Void
Absorbance 280 nm
h
SiA
SiB
SiC
SiD
SiE
+ Fabs
hSERINC3
Void
Absorbance 280 nm
Elution Volume (mls)
c
Void
Absorbance 280 nm
Elution Volume (mls)
d
Void
Absorbance 280 nm
Elution Volume (mls)
i
kDa
75
50
37
25
SERINC3
SiA
SiB
SiC
SiD
SiE
e
kDa
250
150
100
75
50
37
25
20
15
10
Pooled Fractions
SERINC3
SiA
f
kDa
250
150
100
75
50
37
25
20
15
10
Pooled Fractions
SERINC3
ΔICL4
SiA
g
10 mM
BS3
-
+
kDa
250
50
25
10

Supplementary Figure 2. | Purification of WT hSERINC3, ΔICL4-hSERINC3 and formation of complexes with the Fab SiA (Kossiakoff designation). (**a-i**) Shown are the experiments used to generate purified full-length WT hSERINC3 and ΔICL4-hSERINC3 used for the single-particle cryoEM data collections that resulted in the maps shown in **Figures 1** and **Supplementary Fig. 6e.** SEC chromatograms from Superdex200 gel filtration purified hSERINC3 (**a**) or ΔICL4-hSERINC3 (**b**) solubilized in DDM/CHS and exchanged into GDN. Insets show the Simply Blue gels of the purified proteins after elution from the Strep-Tactin beads that were loaded onto the Superdex200 column. Fractions with monomeric hSERINC3 (**c**) or ΔICL4-hSERINC3 (**d**) containing monodisperse protein were then incubated with the Fab SiA and subjected to further gel filtration on the Superdex200 column. The corresponding SEC peaks are shown. Fractions (highlighted in yellow) that were analyzed by SDS-PAGE and silver-stained are shown for hSERINC3-Fab SiA (Experiment replicated ~10x.) (**e**) and ΔICL4-hSERINC3 SiA (Experiment replicated 3x). (**f**). hSERINC3 and ΔICL4-hSERINC3 samples were pooled as indicated (**e,f**), concentrated and used for structure determination. (**g**) The ΔICL4-hSERINC3 sample was cross-linked with 10 mM bis(sulfosuccinimidyl)suberate (BS3) prior to preparation of frozen-hydrated specimens. (**h**) SEC chromatograms displayed a higher molecular weight shift of hSERINC3 in the presence of Fabs designated SiA (red), SiB (green), SiC (blue), SiD (cyan) and SiE (magenta) in 0.02% DDM/CHS in comparison with hSERINC3 alone (black). (**i**) Silver-stained SDS-PAGE fractions across the peaks in (**h**) for hSERINC3 alone and each Fab, n=1 independent replicate. Peak fractions for hSERINC3-Fab (light blue) and hSERINC3 (light red) are boxed in (**h**) and (**i**). Molecular weight standards in (**e**), (**f**), (**i**) and (**g**) are shown to the left. Source data are provided as a Source Data file.

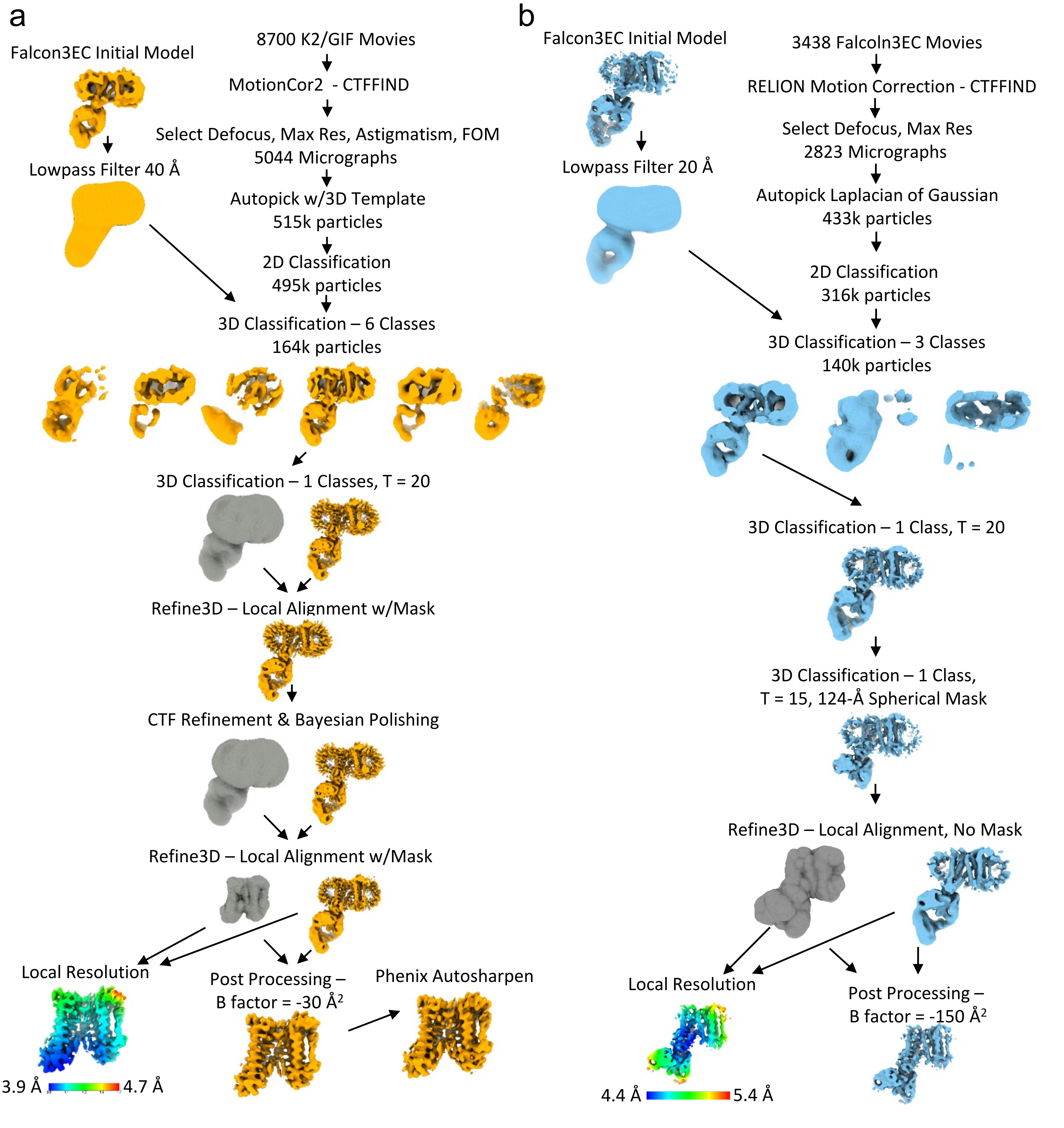

a
Falcon3EC Initial Model
Lowpass Filter 40 Å
8700 K2/GIF Movies
MotionCor2 - CTFFIND
Select Defocus, Max Res, Astigmatism, FOM
5044 Micrographs
Autopick w/3D Template
515k particles
2D Classification
495k particles
3D Classification – 6 Classes
164k particles
3D Classification – 1 Classes, T = 20
Refine3D – Local Alignment w/Mask
CTF Refinement & Bayesian Polishing
Refine3D – Local Alignment w/Mask
Local Resolution
3.9 Å
4.7 Å
Post Processing –
B factor = -30 Å²
Phenix Autosharpen
b
Falcon3EC Initial Model
Lowpass Filter 20 Å
3438 Falcoln3EC Movies
RELION Motion Correction - CTFFIND
Select Defocus, Max Res
2823 Micrographs
Autopick Laplacian of Gaussian
433k particles
2D Classification
316k particles
3D Classification – 3 Classes
140k particles
3D Classification – 1 Class, T = 20
3D Classification – 1 Class,
T = 15, 124-Å Spherical Mask
Refine3D – Local Alignment, No Mask
Local Resolution
4.4 Å
5.4 Å
Post Processing –
B factor = -150 Å²

Supplementary Figure 3. | RELION processing flowcharts of the WT hSERINC3-Fab complex (a, gold) and the ΔICL4-hSERINC3-Fab complex (b, blue). Particles selected from 2D classifications were combined and further processed in 3D. An initial model for 3D had been generated from a dataset collected at the MEMC using the Falcon 3EC direct detector. The final particle set was refined using 3D auto-refine with a mask, followed by CTF refinement and Bayesian polishing, which was then followed by an additional round of 3D auto-refine with a mask. Details of the data processing strategy are described in the Methods section. hSERINC3 ΔICL4-Fab particles selected from 2D classifications were combined and further processed in 3D. An initial model for 3D had been generated from the hSERINC3 ΔICL4-Fab without supplements dataset that had been low-passed filtered to 20 Å. One class from the initial 3D classification was subjected to an additional round of 3D classification (T=20) followed by another round with a 124 Å spherical mask and T=15. 3D refinement with local alignment and without a mask was followed by post-processing with a B-factor of -150 $Å^2$ to generate the final map. Color bars indicate resolution in the final maps.

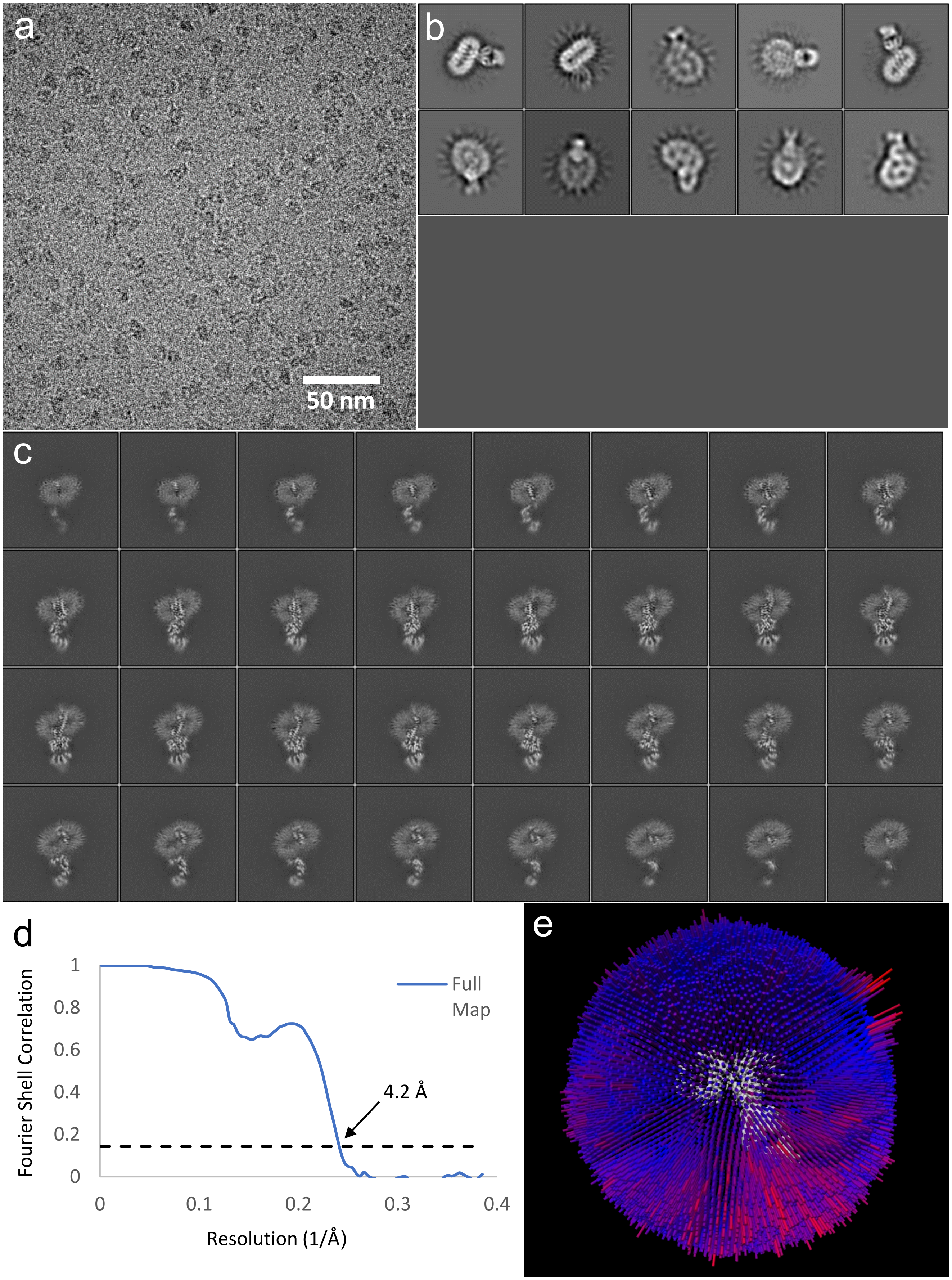
a
50 nm
b
c
d
Fourier Shell Correlation
Resolution (1/Å)
Full Map
4.2 Å
1
0.8
0.6
0.4
0.2
0
0
0.1
0.2
0.3
0.4
e

Supplementary Figure 4. | RELION processing of the wildtype hSERINC3-Fab complex. (**a**) Representative cryoEM image of hSERINC3-Fab complexes displays homogeneous and monodisperse particles. (**b**) 2D class averages of particles from cryoEM images. (**c**) 2D slices through the 3D map. (**d**) FSC (Fourier shell correlation) curve between independently refined half-maps of the final reconstruction of the hSERINC3–Fab complex. Resolution of the final map was evaluated by gsFSC (gold-standard FSC) method where the FSC curve crossed a correlation value of 0.143. (**e**) Euler angle particle distribution of reconstruction. Refer to Statistics and Reproducibility for a discussion of experimental replicates.

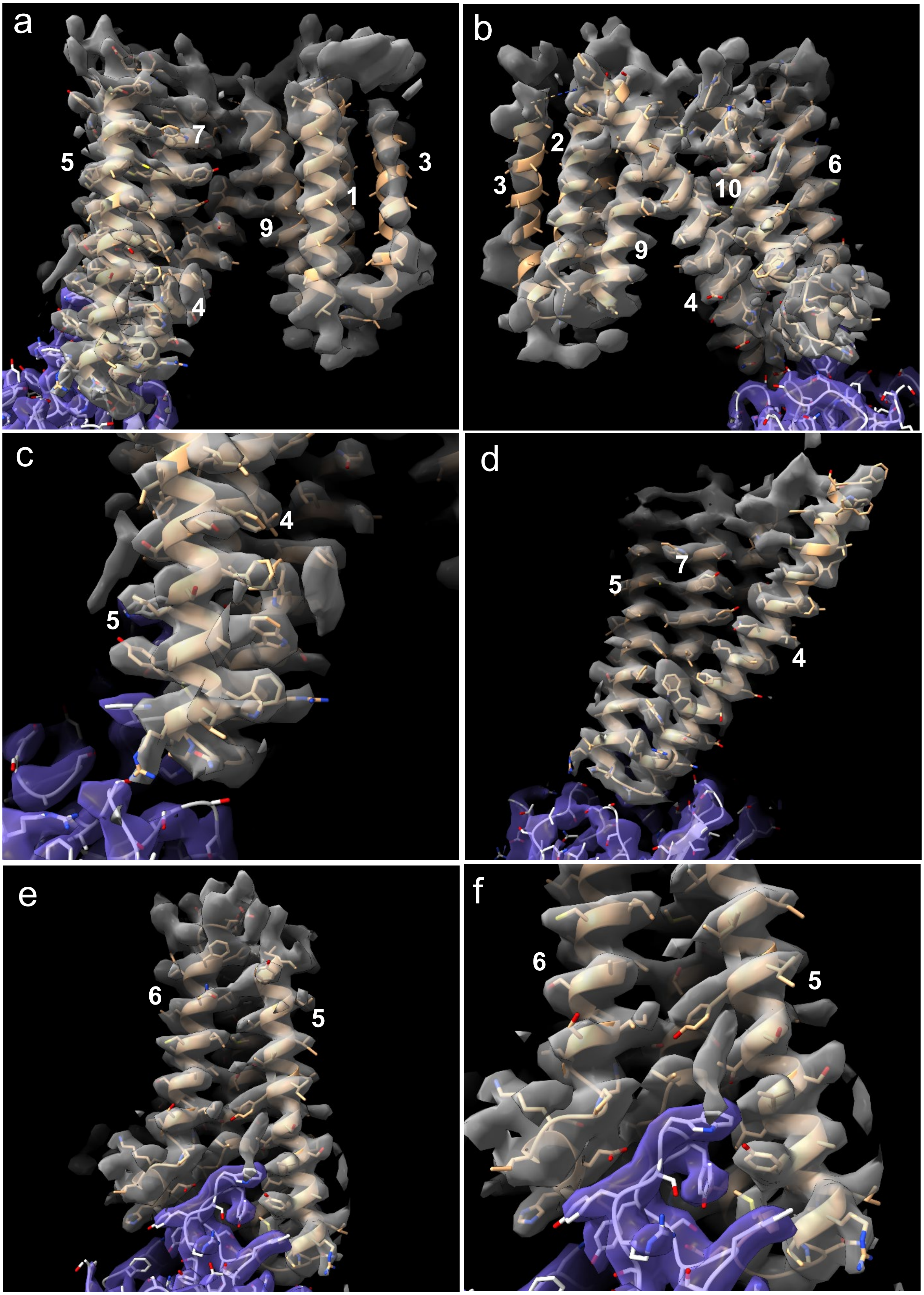

a
5
7
9
1
3
4
b
3
2
10
6
9
4
c
4
5
d
7
5
4
e
6
5
f
6
5

Supplementary Figure 5. | CryoEM density map and refined structure of WT hSERINC3-Fab complexes (α-helices labeled as in Figure 1e). CryoEM density map of WT hSERINC3 (gold) and the variable region of the bound Fab (purple). The cryoEM density map is shown as transparent white (hSERINC3) or transparent purple (Fab), with the fitted model of hSERINC3 (cartoon colored in gold) and Fab (white cartoon). (**a,c**) Map displays two α-helical bundles connected by a highly tilted 40 residue "crossmember" helix. (**a,b**, related by a 180° rotation) (**b**) Counterclockwise rotation by 90° about the vertical and enlargement of the Fab-binding helical bundle from the position in (**a**), emphasizing an oblong density with a cluster of adjacent tyrosine residues. (**d**) Counterclockwise rotation by 90° about the vertical from the position in (**b**) showing the highly tilted 40 residue "crossmember" helix. (**e**) View highlighting the Fab variable region where the CDR loops bind to an hSERINC3 conformational epitope. (**f**) Enlargement of (**e**) showing the aromatic side chains in the CDR loops. The map resolution of the Fab-proximal bundle was ~3.6 Å, with well-defined side chain densities. The resolution of the distal bundle is ~4.4 Å.

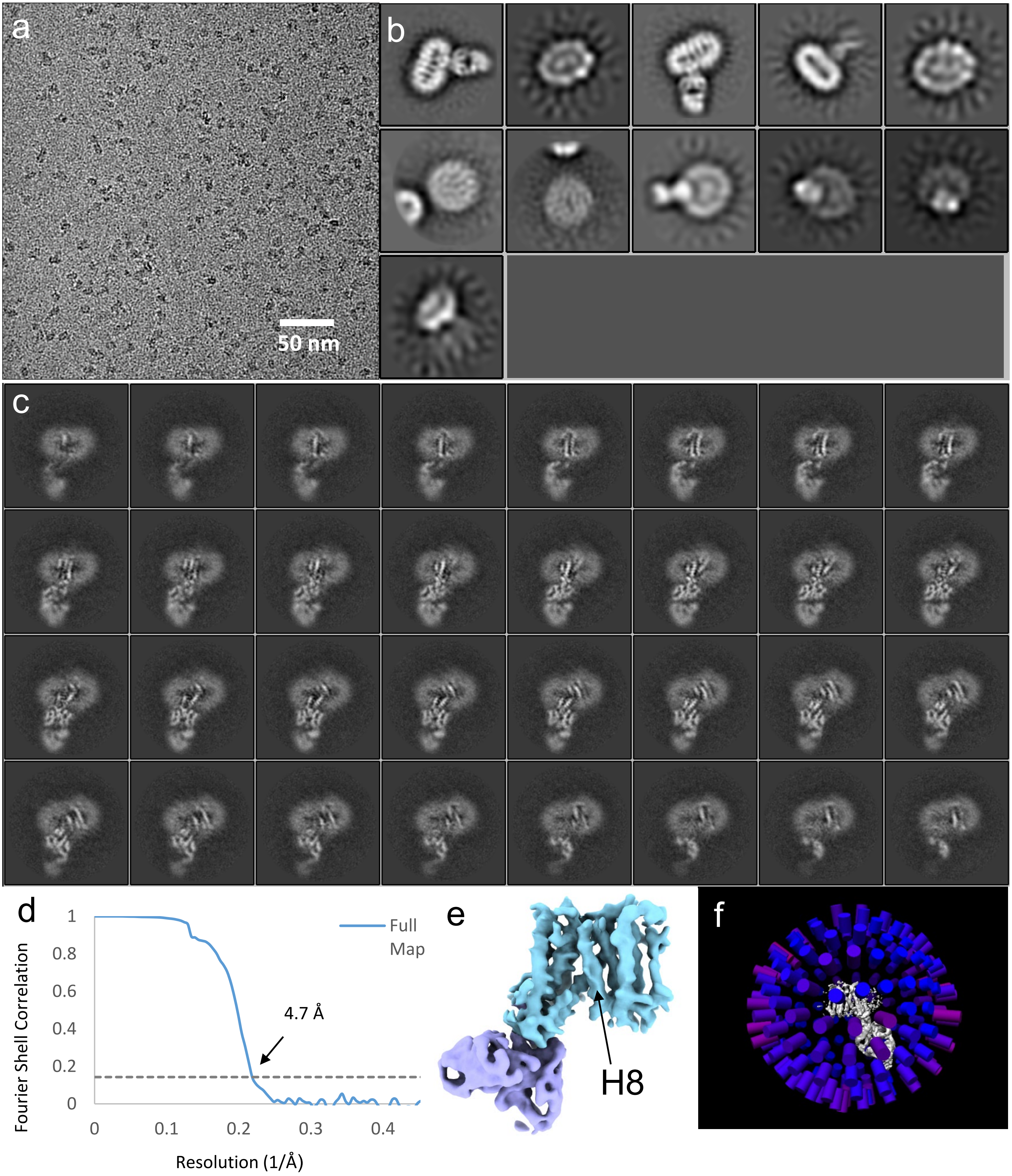

a
50 nm
b
c
d
Fourier Shell Correlation
Resolution (1/Å)
Full Map
4.7 Å
1
0.8
0.6
0.4
0.2
0
0
0.1
0.2
0.3
0.4
e
H8
f

Supplementary Figure 6. | RELION processing of the ΔICL4-hSERINC3-Fab complex. (**a**) Representative cryoEM image of ΔICL4-hSERINC3-Fab complex displays homogeneous and monodisperse particles. (**b**) 2D class averages of particles from cryoEM images. (**c**) 2D slices through the 3D map. (**d**) FSC **(**Fourier shell correlation) curve between independently refined half-maps of the final reconstruction of the ΔICL4-hSERINC3-Fab complex. Resolution of the final map was evaluated by gsFSC (gold-standard FSC) method where the FSC curve crossed a correlation value of 0.143. (**e**) CryoEM map of ΔICL4-hSERINC3 (blue) with a bound Fab (purple) showing stabilization of helix 8. (**f**) Euler angle particle distribution of reconstruction. . Refer to Statistics and Reproducibility for a discussion of experimental replicates.

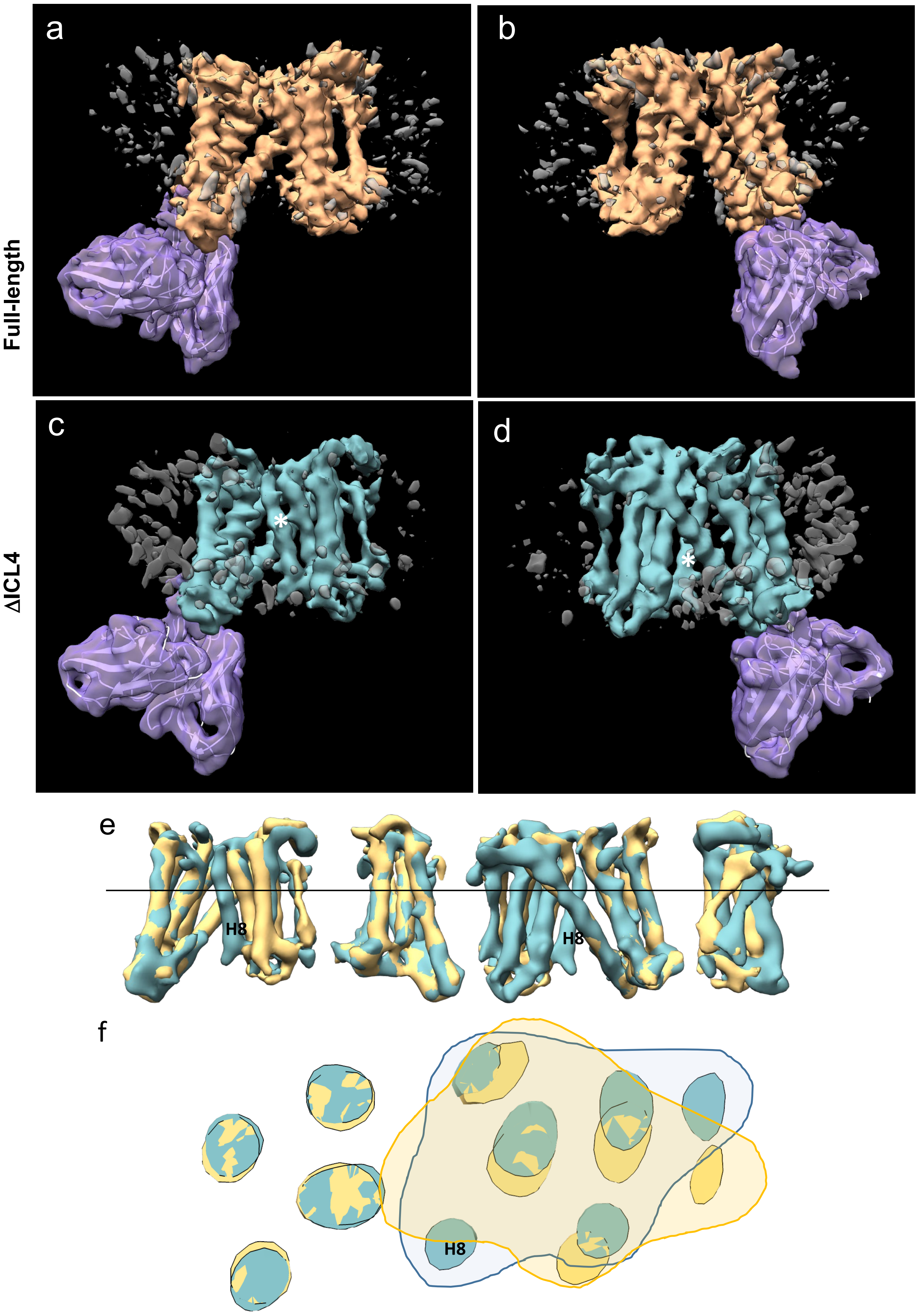

a
b
c
d
e
f
Full-length
ΔICL4
*
*
H8
H8
H8

Supplementary Figure 7. | CryoEM density maps of full-length, WT hSERINC3 (a, b; gold) and a deletion mutant of the fourth intracellular loop (ΔICL4) (c, d; blue) and the variable region of the bound Fab (purple). (**e**) The secondary structure model shows that hSERINC3 is comprised of two α-helical bundles. The proximal Fab-binding bundle contains H5, 6, 7 and 10, and the distal bundle contains H1, 2, 3 and 9. The two bundles are connected by a long 40 residue, diagonal "crossmember" α-helix (H4). H4 is paired with H8, which displays conformational variability in the full-length WT map and is better-ordered in the ΔICL4 deletion mutant (asterisks in (**c**) and (**d**). (**f,g**) Superposition of the cryo-EM density maps of full-length, WT hSERINC3 (gold) and the ΔICL4 deletion mutant (blue). (**f**) Horizontal line in the cross-section view identifies the position of the superimposed *en face* view in (**g**). The ΔICL4 deletion elicits rotation of the less ordered bundle.

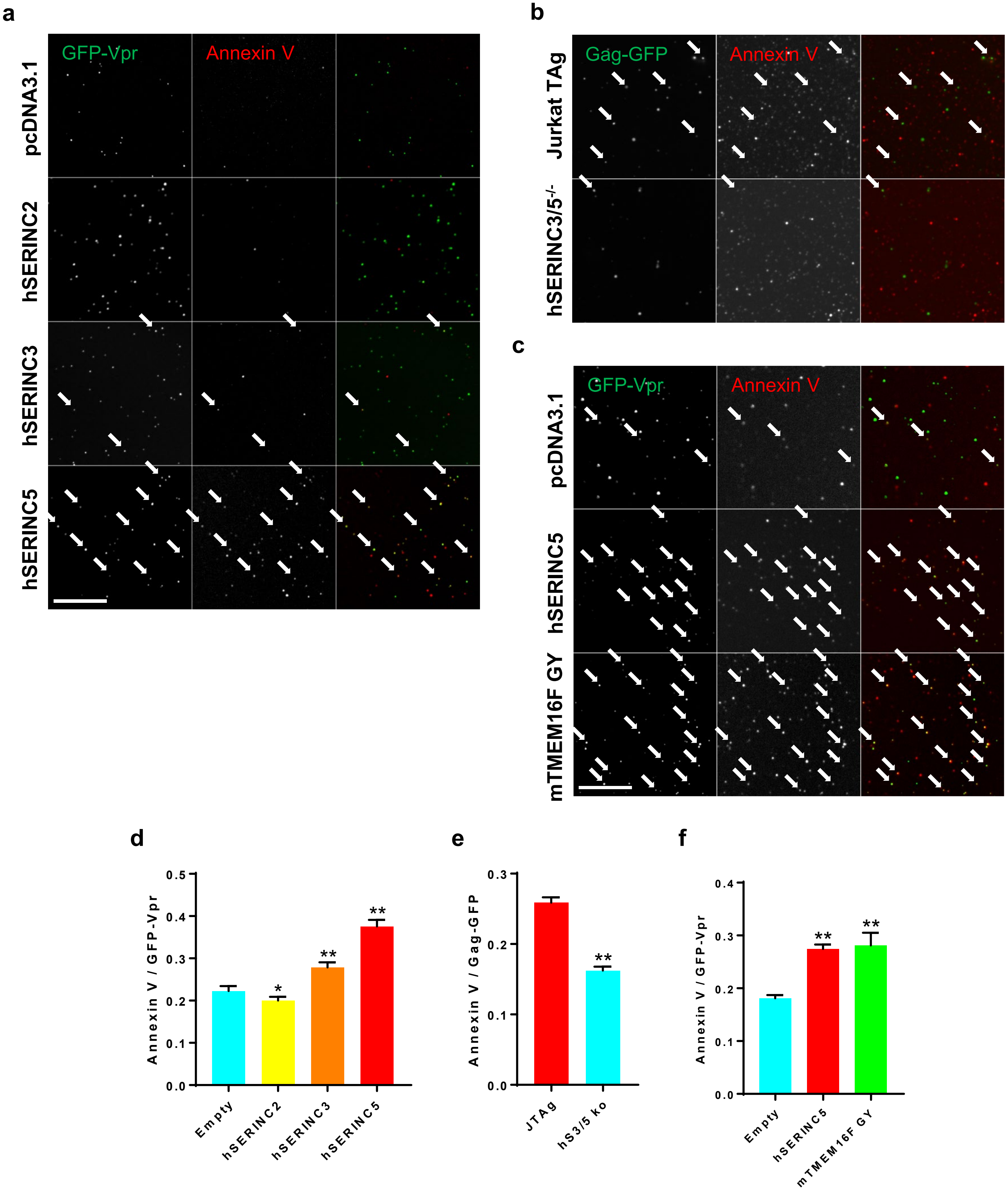
a
GFP-Vpr
Annexin V
pcDNA3.1
hSERINC2
hSERINC3
hSERINC5
b
Gag-GFP
Annexin V
Jurkat TAg
hSERINC3/5-/-
c
GFP-Vpr
Annexin V
pcDNA3.1
hSERINC5
mTMEM16F GY
d
Annexin V / GFP-Vpr
Empty
hSERINC2
hSERINC3
hSERINC5
e
Annexin V / Gag-GFP
JTAg
hS3/5 ko
f
Annexin V / GFP-Vpr
Empty
hSERINC5
mTMEM16F GY

Supplementary Figure 8. | hSERINC3, hSERINC5, and mTMEM16F GY expose PS on the surface of HIV-1 particles. (**a**) HEK293-derived HIV-1_{NL4-3}ΔRTΔNef virus particles containing GFP-Vpr were produced in the presence of the indicated hSERINC plasmid, immobilized on poly-lysine coated coverslips and stained for PS exposure using Alexa594 annexin V. (**b**) HIV-1_{NL4-3}ΔNef virus particles containing Gag-EGFP were produced from parental or hSERINC3/5 knockout Jurkat TAg cells, immobilized on poly-lysine coated coverslips, and stained with Alexa594 annexin V, prior to fixation with 4% PFA, and analysis by confocal microscopy as in (**a**). (**c**) HIV-1_{NL4-3}ΔRTΔNef virus particles containing GFP-Vpr were produced in the presence of WT hSERINC5 or mTMEM16F GY plasmid, stained, and analyzed as in (**a**)**.** (**d-f**) Mean annexin V fluorescence for individual HIV-1 particles was quantified and normalized to GFP intensity. Particles (n=400) were quantified per condition, from n=2 independent experiments. Scalebar = 5 mm. Values represent mean annexin V intensity per GFP-Vpr or Gag-GFP intensity ± SEM. Significance was determined using a one-way ANOVA test. P values are as follows: (**d**) hSERINC2 p=0.015, hSERINC3 p<0.0001, hSERINC5 p<0.0001 (**e**) hS3/5ko p<0.0001 (**f**) hSERINC5 p<0.0001, mTMEM16FGY p<0.0001. Source data are provided as a Source Data file.

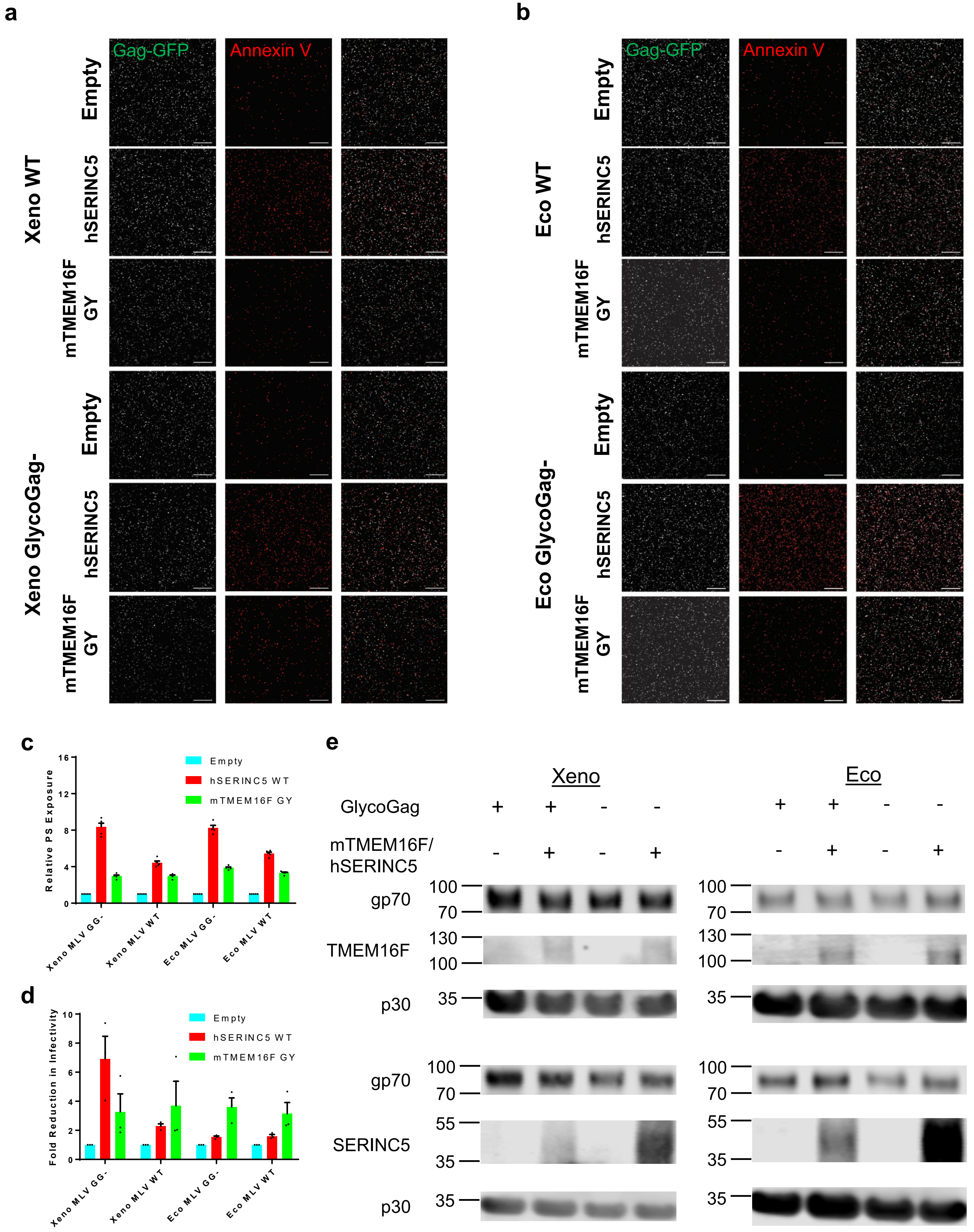

a
b
Gag-GFP
Annexin V
Empty
hSERINC5
mTMEM16F GY
Xeno WT
Xeno GlycoGag-
Eco WT
Eco GlycoGag-
c
Relative PS Exposure
Empty
hSERINC5 WT
mTMEM16F GY
Xeno MLV GG-
Xeno MLV WT
Eco MLV GG-
Eco MLV WT
d
Fold Reduction in Infectivity
e
Xeno
Eco
GlycoGag
mTMEM16F/
hSERINC5
gp70
TMEM16F
p30
SERINC5

Supplemental Figure 9 | hSERINC5 and mTMEM16F GY expose PS on the surface of MLV particles. (**a**) MLV VLPs containing GagGFP and Xenotropic MLV Env with or without GlycoGag were produced in the presence of the indicated plasmid. VLPs were immobilized on poly-lysine coated coverslips, stained with Alexa594 annexin V, and imaged by confocal microscopy. (**b**) MLV VLPs containing Eco MLV Env were produced and analyzed as in (**a**). Scalebar = 10 mm. (**c**) Percent annexin V-positive VLPs were quantified using CellProfiler. Values represent the mean of n=5 independent experiments ± SEM. (**d**) Relative infectivity of viruses used in (**a**) and (**b**) was determined by firefly luciferase assay in HT1080-mCAT1 cells. Values shown represent the mean of n=3 independent experiments ± SEM. Scalebar = 10mm. (**e**) MLV VLPs were produced as in (**a**) and (**b**), lysed, and subjected to SDS-PAGE and Western blotting for MLV Env (gp70), MLV CA (p30), hSERINC5 (using anti-SERINC5), and mTMEM16F GY (using anti-FLAG). Xeno – xenotropic MLV Env, Eco – ecotropic MLV Env. Source data are provided as a Source Data file.

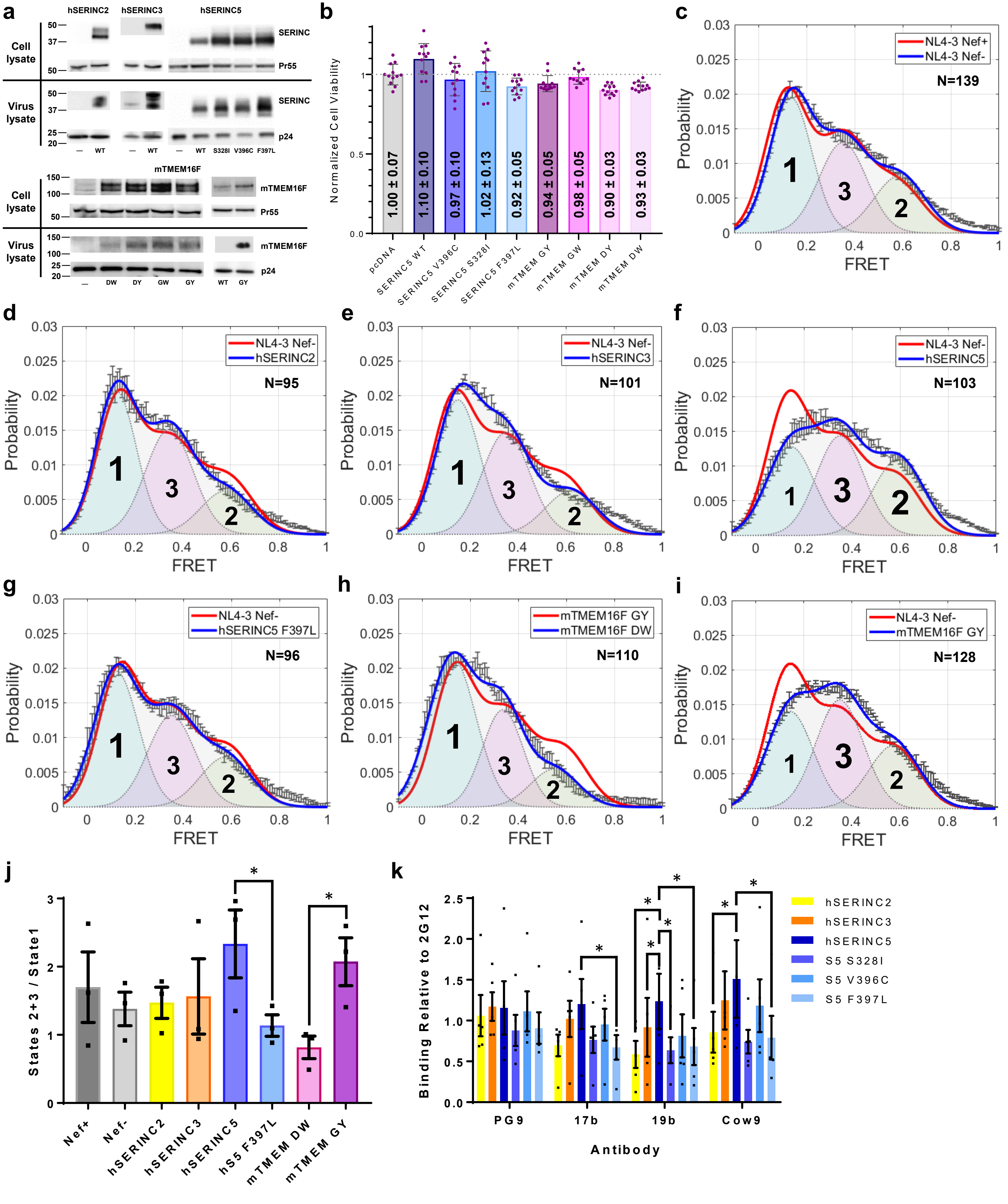

a
hSERINC2
hSERINC3
hSERINC5
Cell lysate
Virus lysate
SERINC
Pr55
p24
WT
S328I
V396C
F397L
mTMEM16F
DW
DY
GW
GY
b
Normalized Cell Viability
1.00 ± 0.07
1.10 ± 0.10
0.97 ± 0.10
1.02 ± 0.13
0.92 ± 0.05
0.94 ± 0.05
0.98 ± 0.05
0.90 ± 0.03
0.93 ± 0.03
pcDNA
SERINC5 WT
SERINC5 V396C
SERINC5 S328I
SERINC5 F397L
mTMEM GY
mTMEM GW
mTMEM DY
mTMEM DW
c
NL4-3 Nef+
NL4-3 Nef-
N=139
Probability
FRET
d
NL4-3 Nef-
hSERINC2
N=95
e
NL4-3 Nef-
hSERINC3
N=101
f
NL4-3 Nef-
hSERINC5
N=103
g
NL4-3 Nef-
hSERINC5 F397L
N=96
h
mTMEM16F GY
mTMEM16F DW
N=110
i
NL4-3 Nef-
mTMEM16F GY
N=128
j
States 2+3 / State1
Nef+
Nef-
hSERINC2
hSERINC3
hSERINC5
hS5 F397L
mTMEM DW
mTMEM GY
k
Binding Relative to 2G12
hSERINC2
hSERINC3
hSERINC5
S5 S328I
S5 V396C
S5 F397L
PG9
17b
19b
Cow9
Antibody

Supplementary Figure 10. | hSERINC5 and mTMEM16F GY expression promote opening of the HIV-1 Env trimer. (**a**) HIV-1_{NL4-3}ΔNef viruses were produced in the presence of indicated hSERINC/mTMEM16F plasmid. Cell and virus lysates were subjected to SDS-PAGE and Western immunoblotting for HIV-1 Gag (Pr55), HIV-1 CA (p24), and the indicated hSERINC/mTMEM16F protein using anti-FLAG or anti-SERINC3 antibodies. (**b**) HEK293 cells were transfected with HIV-1_{NL4-3}ΔRTΔNef and the indicated hSERINC5/mTMEM16F plasmid. Cell viability was assessed by measuring the total ATP content of freshly-prepared cell lysates using a commercially available kit. Data shown represent mean ± SD from n=3 independent experiments. (**c-i**) HIV-1_{NL4-3}ΔRTΔNef virus particles were produced in the presence of the indicated hSERINC/mTMEM16F plasmid and analyzed by single-molecule Förster-resonance energy transfer (smFRET). Histograms in panels (**d**) to (**i**) are shown compared to Nef-. Conformational states are designated as State 1 (pre-triggered, closed conformation), State 2 (necessary intermediate conformation), and State 3 (fully-open, CD4-bound conformation) The total number of raw FRET traces used in each histogram is shown as N). Histogram error bars represent the mean FRET probabilities ± SEM. (**j**) Occupancies for each conformational state were calculated as the area under each curve. The ratio of open to closed Env trimers is displayed as the ratio of (State 2 + State 3) / State 1. Values shown represent the mean of n=3 subsets of single-molecule trajectories ± SEM. P values were calculated using a one-tailed, unpaired t test. Asterisks identify statistically significant ($p<0.05$) differences in the comparisons. hSERINC5 > hS5 F397L (p=0.04), mTMEM16F GY > mTMEM DW (p=0.02). (**k**) HIV-1_{NL4-3} VLPs were prepared and subjected to virus capture assay (VCA) using the indicated antibody normalized to 2G12. Values represent the mean of n=5 independent experiments ± SEM. Statistical significance was determined by one-way ANOVA test. Asterisks identify statistically significant ($p<0.05$) differences in the comparisons: 17b Antibody: WT hSERINC5 > S328I (p=0.05), and F397L (p=0.03); 19b Antibody: WT hSERINC5 > WT hSERINC3 (p=0.01), WT hSERINC2 (p=0.03), hSERINC5 S328I (p=0.03) and F397L (p=0.01); Cow9 Antibody: WT hSERINC5 > WT hSERINC2 (p=0.05), and hSERINC5 F397L (p=0.04). Source data are provided as a Source data file.

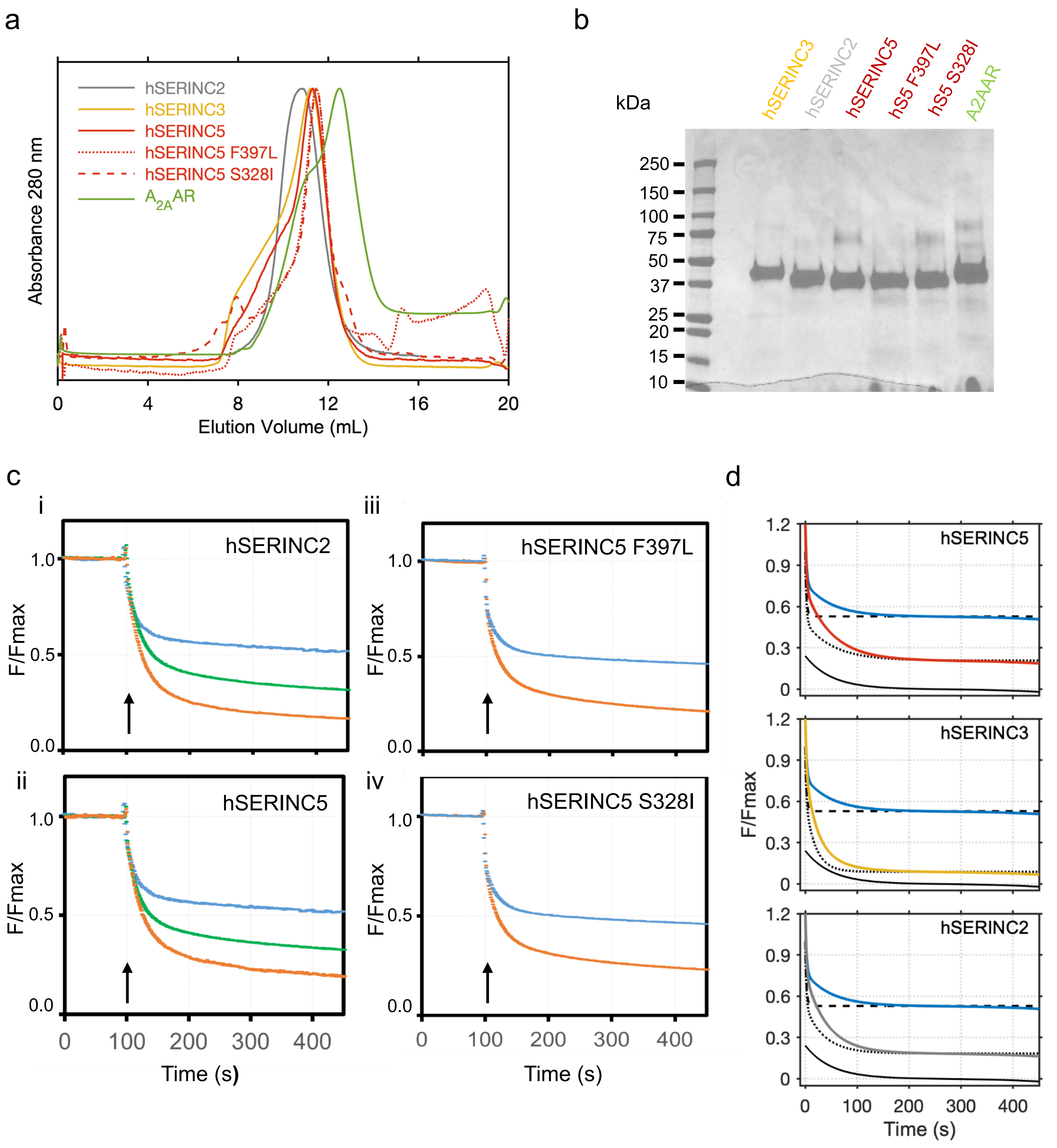

a
hSERINC2
hSERINC3
hSERINC5
hSERINC5 F397L
hSERINC5 S328I
A2AAR
Absorbance 280 nm
0
4
8
12
16
20
Elution Volume (mL)
b
kDa
hSERINC3
hSERINC2
hSERINC5
hS5 F397L
hS5 S328I
A2AAR
250
150
100
75
50
37
25
20
15
10
c
i
hSERINC2
ii
hSERINC5
iii
hSERINC5 F397L
iv
hSERINC5 S328I
F/Fmax
1.0
0.5
0.0
0
100
200
300
400
Time (s)
d
hSERINC5
hSERINC3
hSERINC2
F/Fmax
1.2
0.9
0.6
0.3
0
0
100
200
300
400
Time (s)

Supplementary Figure 11. | Purification and lipid flipping activity of hSERINC 3 (Figure 2), hSERINC5, hSERINC2, hSERINC5 mutants F397L and S328I, and A_{2A}AR (Figure 2). (**a**) Representative SEC chromatograms from Superdex 200 gel filtration of purified hSERINC2, hSERINC3 (Figure 2), hSERINC5, hSERINC5-F397L, hSERINC5-S328I and A_{2A}AR in DDM/CHS. (Each purification was performed 5 to 10x). For hSERINC2, the chromatogram is from a second gel filtration step used to further purify hSERINC2 monomers. (**b**) Simultaneous SDS-PAGE of the purified proteins stained by Simply Blue (performed 2x). (**c**) Representative fluorescence traces (n=3 independent experiments) corresponding to dithionite treatment of NBD-PC for empty liposomes (blue) or proteoliposomes containing (i) hSERINC2 and (ii) hSERINC5, and the mutants (iii) hSERINC5 F397L and (iv) hSERINC5 S328I. The orange curves are at a protein concentration of 1.5 µg/mg of lipid, and the green curves in (i.) and (ii.) are at 0.5 µg/mg of lipid (green). Arrows indicate the addition of dithionite at 100s. (**d**) Representative traces of exponential fits to fluorescent decay data for hSERINCs 5, 3 and 2, such as those shown in (**c**) panels (i) and (ii) and **Figure 2c**. See Statistics and Reproducibility for details. Source data are provided as a Source Data file.

	WT hSERINC3-* Fab	ΔICL4-hSERINC3-# Fab + BS3
**Data collection and processing**		
Voltage (kV)	300	300
Energy filter (eV)	20	
Electron exposure (e- /Å^2)	44	58
Defocus range (- µm)	0.8 - 1.5	1.0 - 2.2
Pixel size (Å)	0.56	0.67
Total micrograph movies	8710	1574
Selected micrograph movies	5044	1418
Initial particle images (no.)	495,000	247,000
Final particle images (no.)	164,000	121,000
Map resolution (Å) (Fab-proximal/Fab-distal)	4.2 (3.6/4.4)	5.1
Map sharpening B factor (Å^2)	-30	-150
FSC threshold	0.143	0.143
**Refinement**		
Refinement resolution (Å)	3.7	4.0
Model composition		
Protein residues	303	302
Nonhydrogen atoms	4859	4940
B factors (Å^2) (min - max)	147 (106 - 187)	262 (184 - 364)
**Protein**		
r.m.s. deviations		
Bond lengths (Å)	0.004	0.011
Bond angles (°)	0.8	1.3
**Validation**		
MolProbity score	1.53	1.55
Clashscore	2.68	3.85
Poor rotamers (%)	0	0.37
Ramachandran		
Favored (%)	92.2	94.5
Allowed (%)	9.7	5.5
Disallowed (%)	0	0

Supplementary Table 1. | CryoEM data collection, refinement, and validation statistics. Summary of relevant parameters used during cryoEM data collection and processing. Refinement and validation statistics are provided for the molecular model of WT hSERINC3-Fab and ΔICL4-hSERINC3-Fab crosslinked with BS3. *Data collected at NCCAT; # data collected at the UVa Molecular Electron Microscopy Core.

Uncropped blots

Supplementary Figure 2i

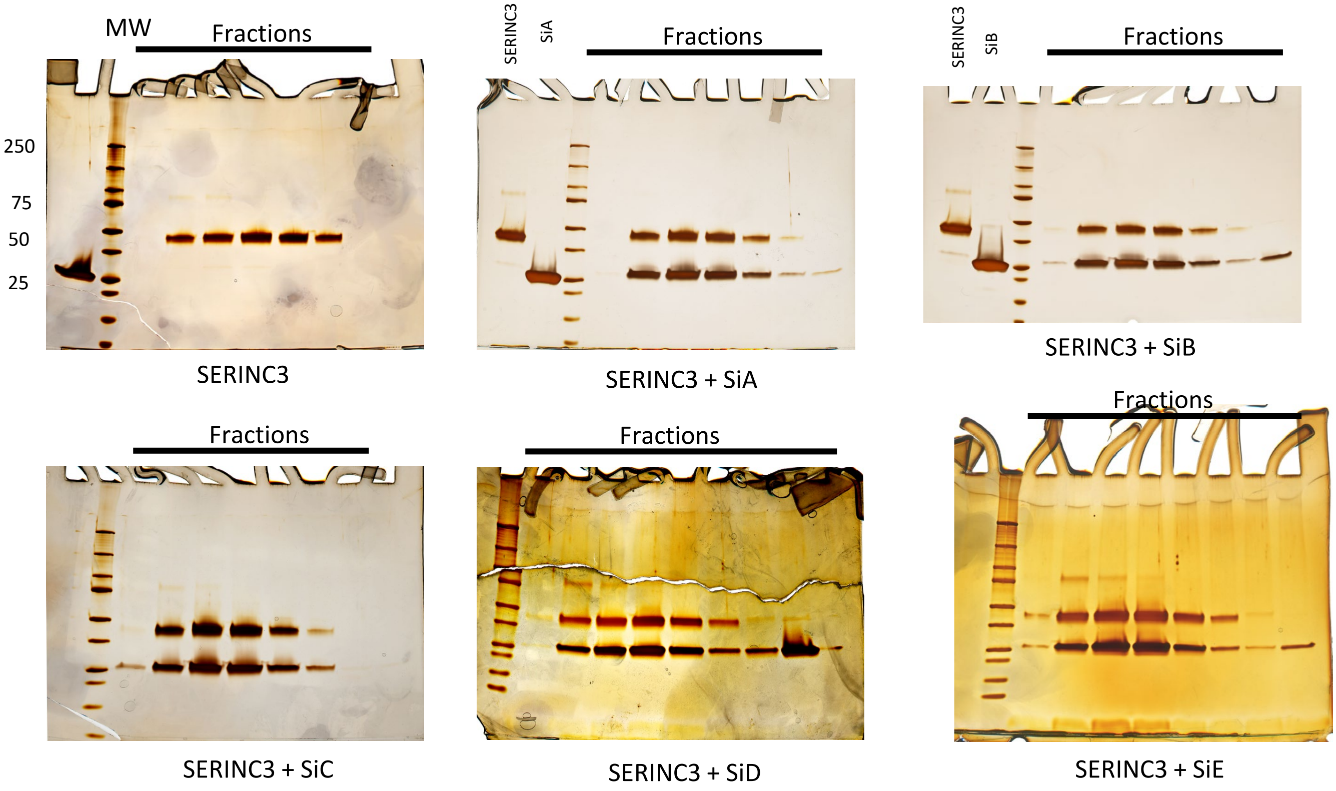

Supplementary Figure 9E

Xeno MLV

mTMEM16F

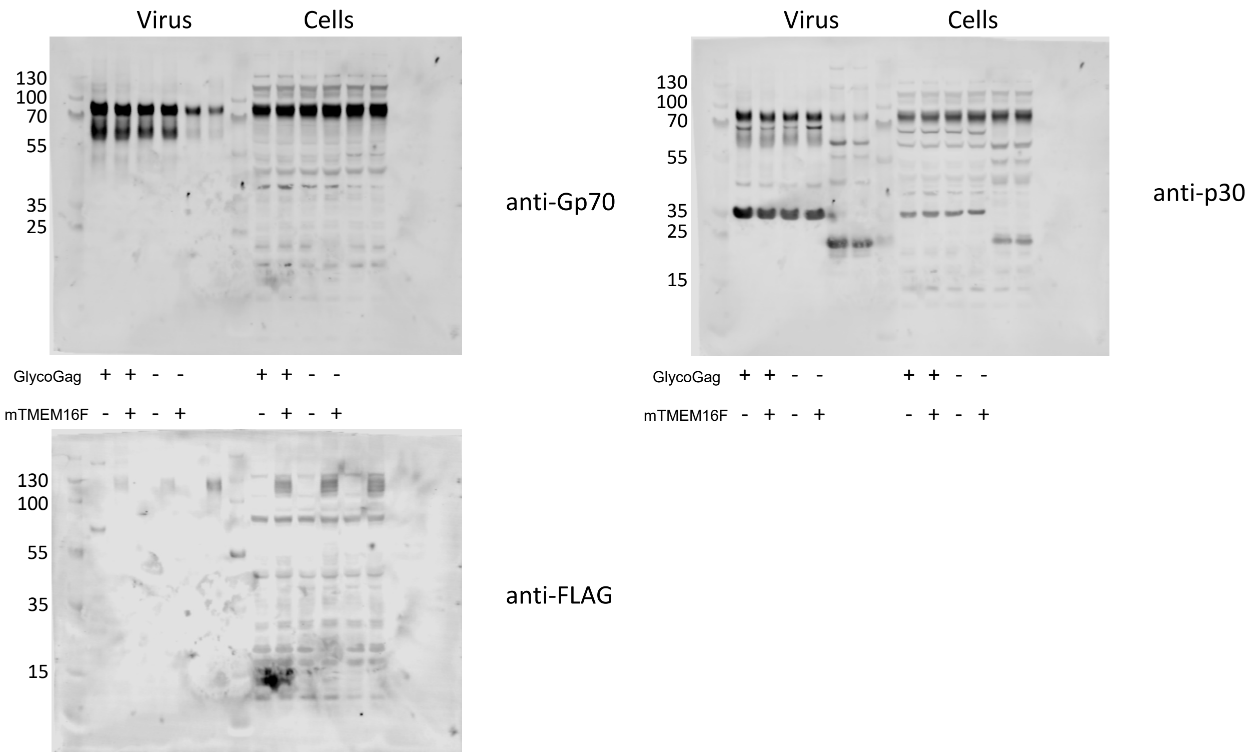

Supplementary Figure 9E

Xeno MLV
hSERINC5

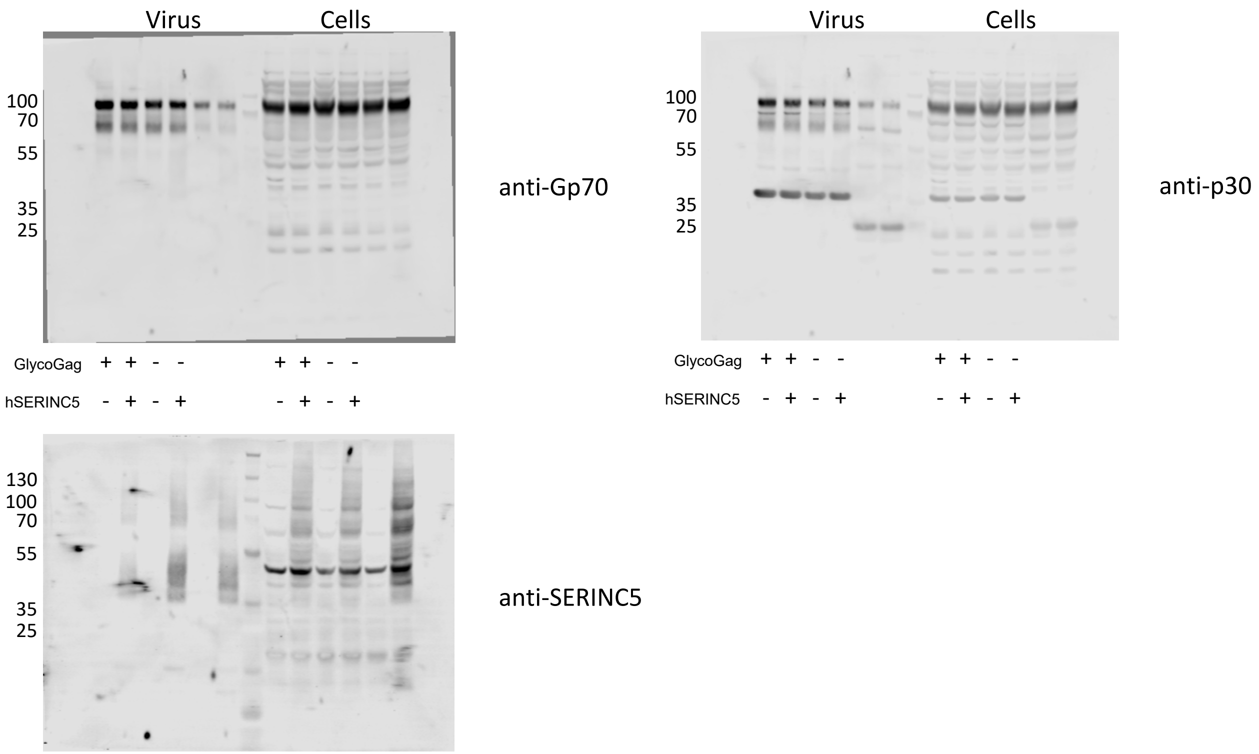

Supplementary Figure 9E

Eco MLV
mTMEM16F

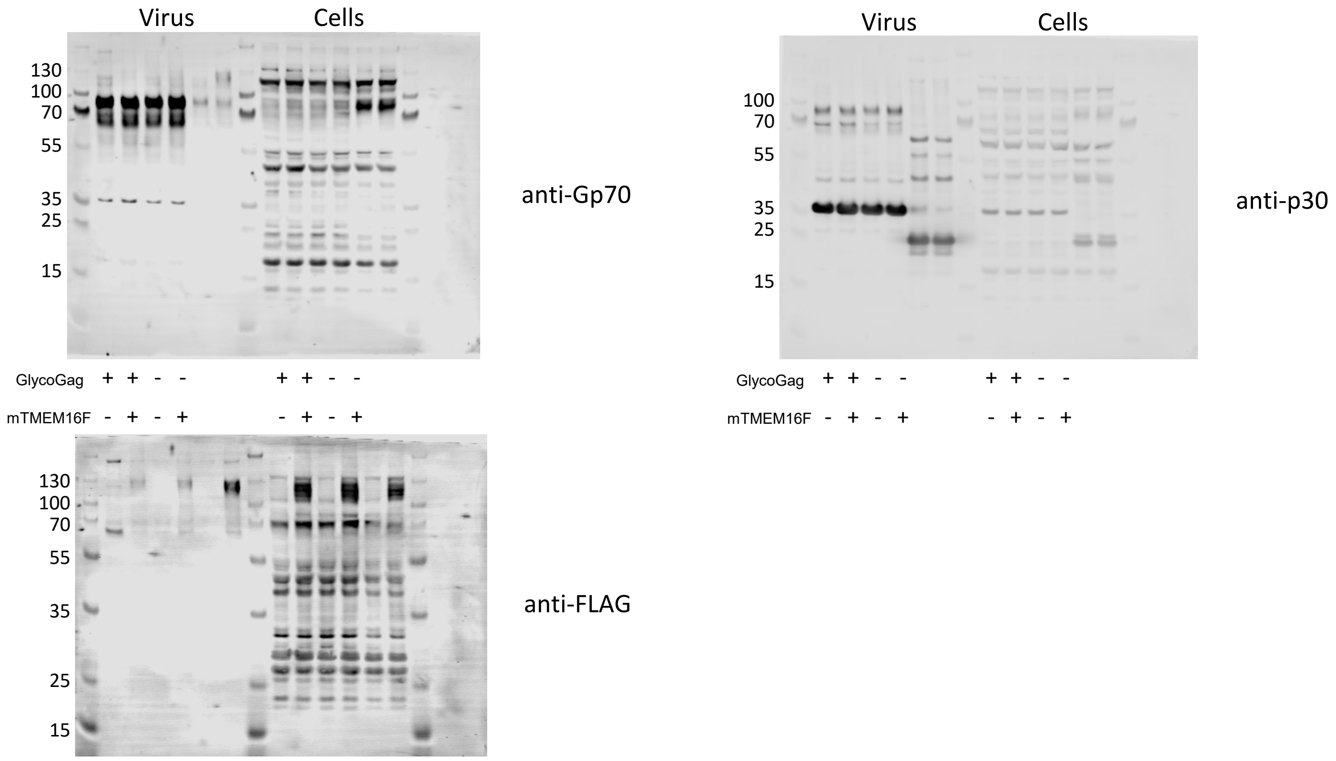

Supplementary Figure 9E

Eco MLV
hSERINC5

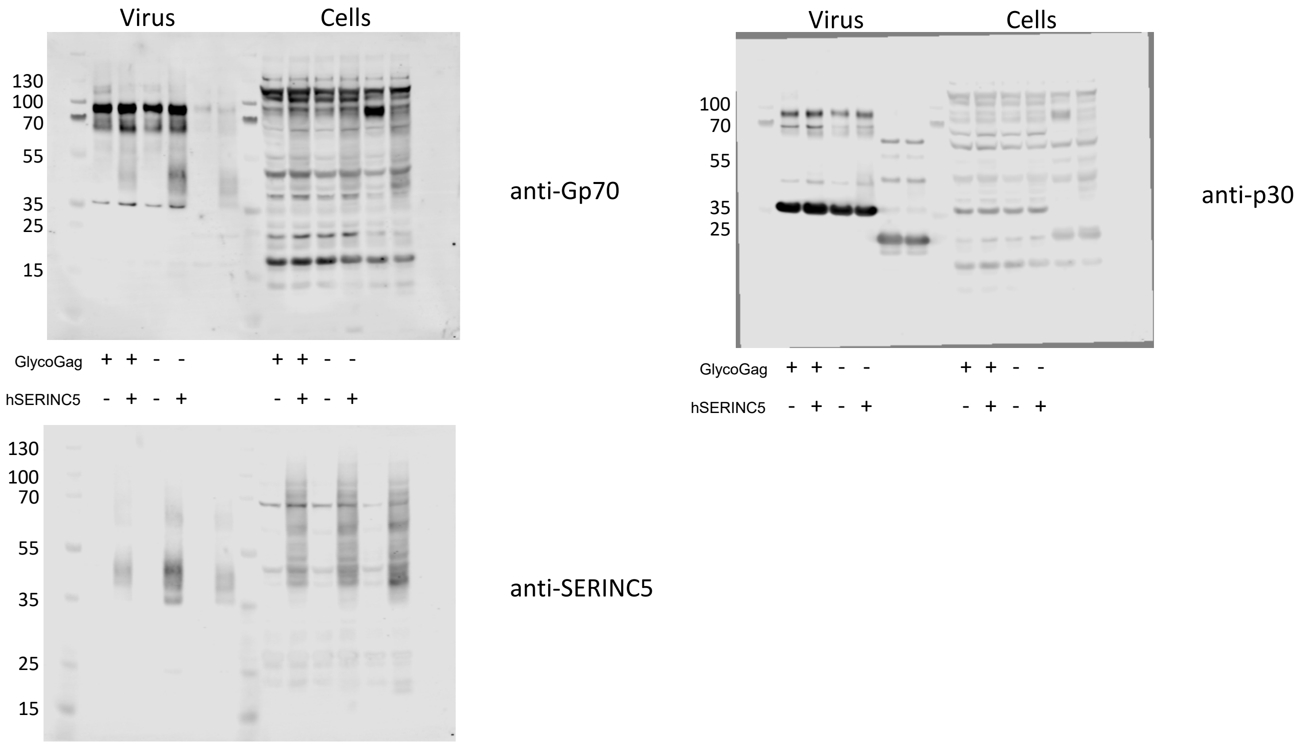

Supplementary Figure 10A

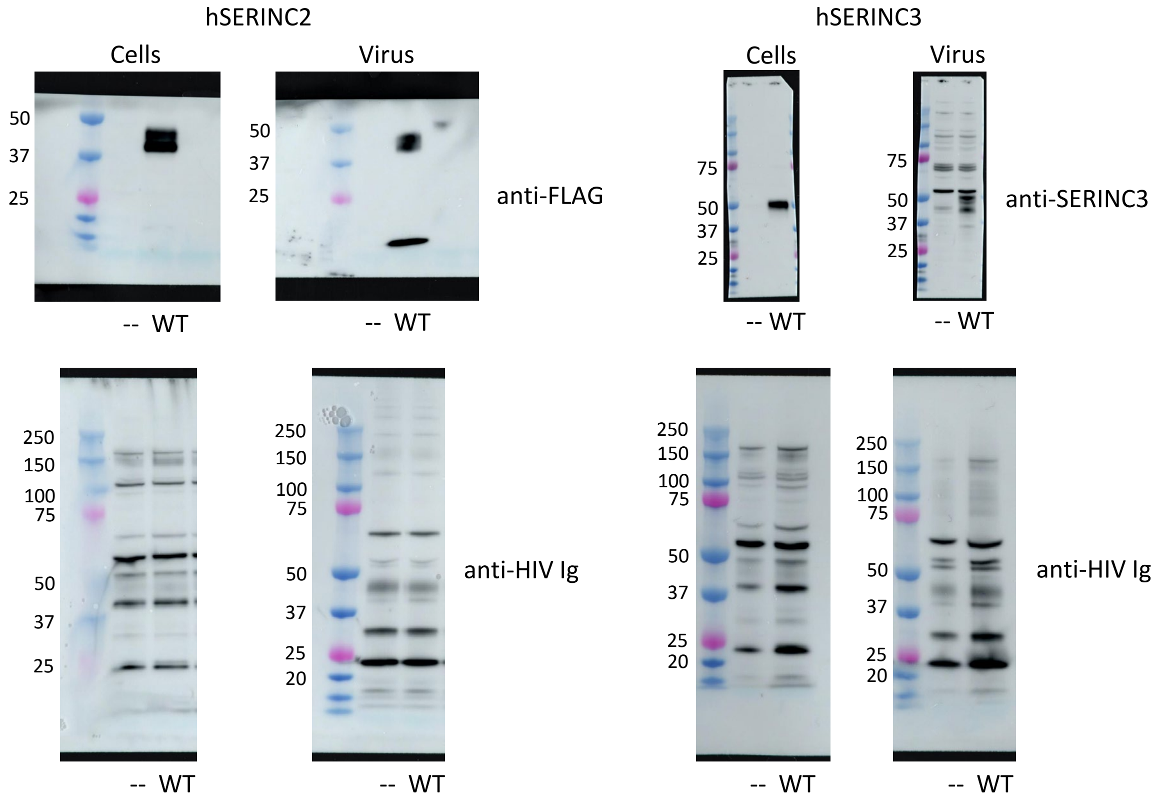

Supplementary Figure 10A

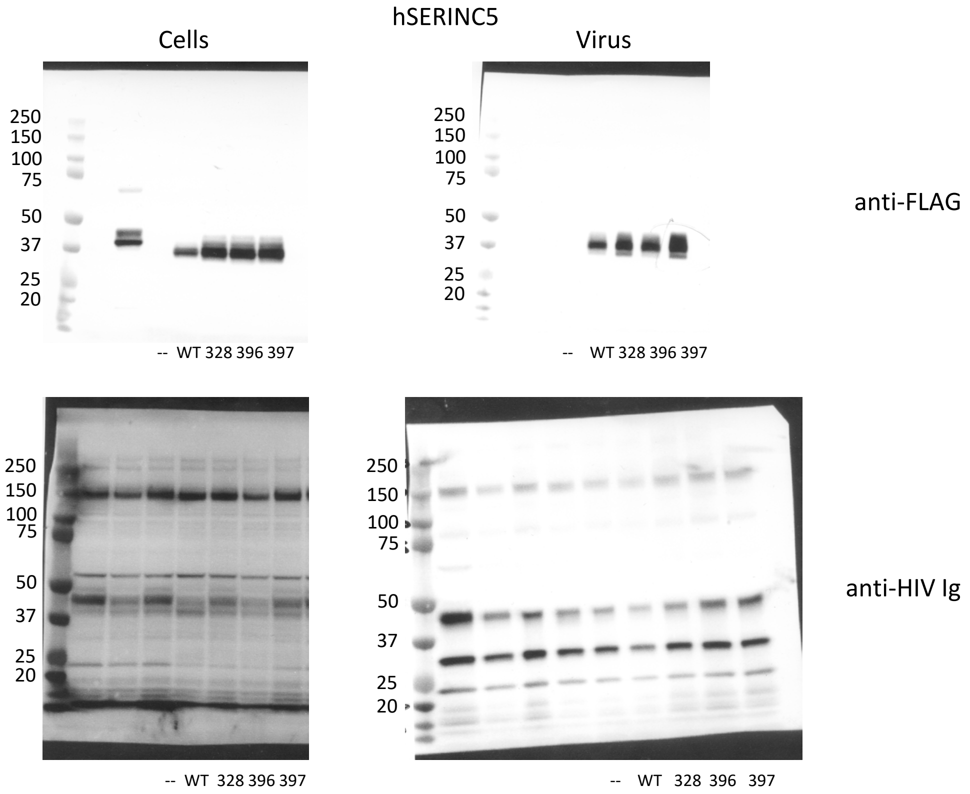

Supplementary Figure 10A

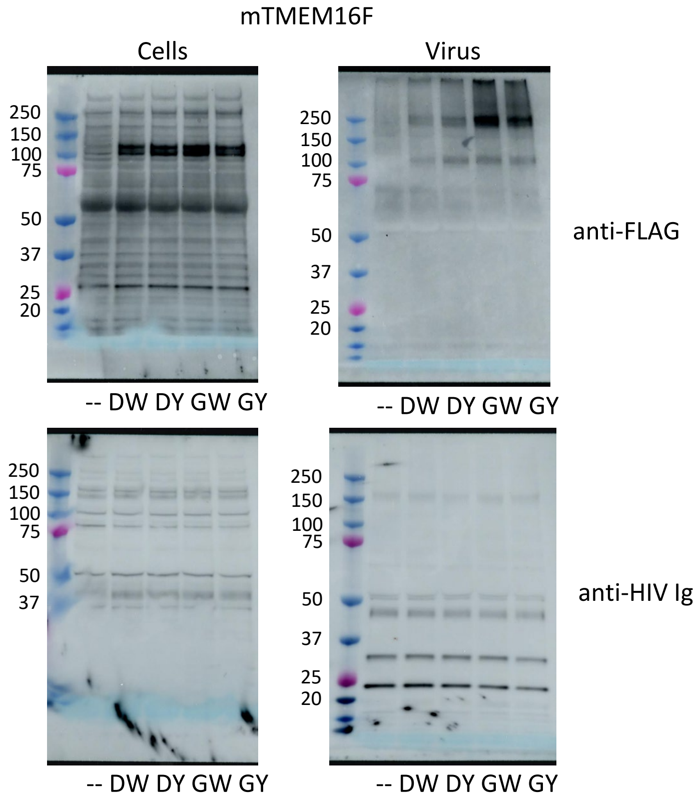

Supplementary Figure 10A

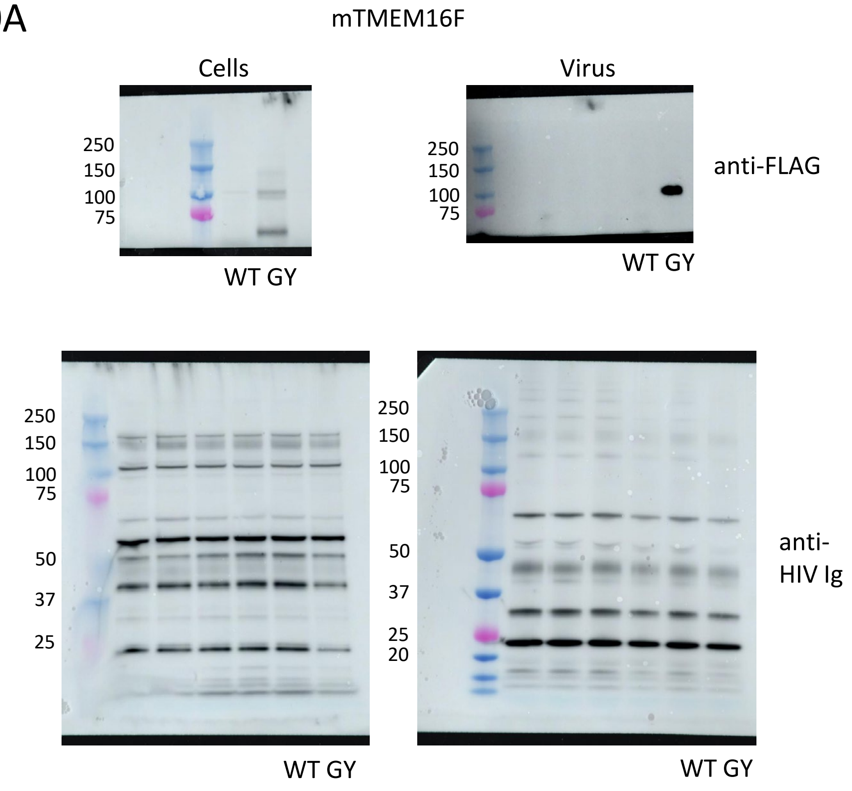

Supplement: Supplementary file 1 — Supplementary Information [file 41467_2023_39262_MOESM1_ESM.pdf]
