# Supplementary material for: Antiviral HIV-1 SERINC restriction factors disrupt virus membrane asymmetry
Source: Nat Commun. 2023 Jul 20;14:4368. doi: 10.1038/s41467-023-39262-2 (PMC10359404; doi:10.1038/s41467-023-39262-2)
Supplement: Supplementary file 3 — Description of Additional Supplementary Files [file 41467_2023_39262_MOESM3_ESM.pdf]

File name: Supplementary Movie 1
Description: The top two SERINC5 AlphaFold models suggest a possible alternating access structural rearrangement for lipid flipping. The movie shows the view from within the lipid bilayer of the conformations predicted by the top two models. The transmembrane α-helices are colored as in **Fig. 1c-e**.

File name: Supplementary Movie 2
Description: The top two SERINC5 AlphaFold models suggest a possible alternating access structural rearrangement for lipid flipping. The movie shows the extracellular view of the conformations predicted by the top two models. The transmembrane α-helices are colored as in **Fig. 1c-e**.
